# Advances in epilepsy surgery

**DOI:** 10.1136/jnnp-2013-307069

**Published:** 2014-04-09

**Authors:** Mark Nowell, Anna Miserocchi, Andrew W McEvoy, John S Duncan

**Affiliations:** 1 Department of Clinical and Experimental Epilepsy, UCL Institute of Neurology, London, UK; 2 MRI Unit, Epilepsy Society, Chalfont St Peter, UK; 3 Department of Neurosurgery, National Hospital for Neurology and Neurosurgery, London, UK

**Keywords:** EPILEPSY, SURGERY, NEUROSURGERY

## Abstract

This review summarises exciting recent and forthcoming advances that will impact on the surgical management of epilepsy in the near future. This does not cover the current accepted diagnostic methodologies or surgical treatments that are routinely practiced today. The content of this review was derived from a PubMed literature search, using the key words ‘Epilepsy Surgery’, ‘Neuromodulation’, ‘Neuroablation’, ‘Advances’, between 2010 and November 2013.

## Introduction

Despite advances in imaging and the accumulation of neurological and surgical experience, the outcomes for seizure freedom in epilepsy surgery have not changed significantly over the last 20 years. Currently, 20–40% of patients with epilepsy are considered refractory to medical treatment.^1^ Less than 50% of these are candidates for focal resective surgery, with rates of long-term seizure freedom ranging from 30% to 60% depending on the operation.^2^ Some argue that this apparent lack of progress is a reflection of a lowered threshold to offer surgery, and that with continued refinement of techniques, increasingly challenging cases are being taken on. However, there is widespread agreement that there remains great potential to improve non-pharmacological management, to achieve either better seizure control or complete seizure freedom.

There are three broad directions in which the next major advances may occur. First, there is the continued refinement of the current methodology. An improved hypothesis for the epileptogenic zone (EZ), based on advanced presurgical evaluation, including intracranial EEG, and thus, better patient selection for cortical resections. This is probably most relevant to sufferers of non-lesional extratemporal epilepsy, and is most likely to lead to improvements in the rates of seizure freedom. Next, there are improved surgical methods for achieving a precisely targeted cortical or subcortical resection. These can be grouped as neuroablative techniques, and include disconnection of propagation pathways, and destruction of epileptogenic foci. Neuroablation may be applied in the treatment of focal and generalised epilepsy. Third, neuromodulation may take a broader role, with the possibility of improving quality of life and being a useful palliation. This is most applicable to patients who are currently not candidates for resective epilepsy surgery, because their epilepsy arises from eloquent cortex, is multifocal or generalised.

## Refinement of current methodology

### The epileptogenic index

The purpose of presurgical evaluation is to define the EZ, and to define the surrounding functional deficit zones. The notion of a single discrete area of EZ is attractive in its simplicity, although the high failure rate of resective surgery runs counter to this. Rather, it is possible that in at least some cases there are several structures involved in epileptogenesis, and a more comprehensive notion of the EZ needs to be considered.

The characteristic electrophysiological pattern of the EZ is the presence of high-frequency oscillations or ‘rapid discharges’. Bartolomei in 2008 introduced the concept of the ‘epileptogenic index (EI)’, a novel quantitative measure that characterises the epileptogenicity of brain structures recorded with depth electrodes.^3^ The EI is based on spectral and temporal factors, with statistically high values corresponding to structures involved early in the ictal process. They found that their measure of EI effectively distinguished between mesial temporal lobe epilepsy (MTLE) and lateral neocortical epilepsy. Furthermore, in MTLE they found a statistically significant correlation between the duration of epilepsy and the number of structures disclosing high EI values, suggesting that MTLE is a gradually evolving process which progresses over time.

Visualisation of the EI, in 3D space as part of an integrated multimodal model, is the obvious next step. This would provide an alternative to the electrical source imaging, but also provide a more detailed, contoured brain map of seizure likelihood, that could be used by the surgeon to stratify the risk-benefit ratio of cortical resection. Work is already underway to make this a reality. David *et al* in Grenoble, report the use of statistical parametric mapping to visualise a quantification of the seizure onset zone.^4^ This has been applied to case studies of insular epilepsy, and also group studies on MTLE. This technique offers much promise in future research and clinical practice, with obvious applications in future guidance of stereoEEG (SEEG) implantations, and in the delineation of epileptogenic networks over time.

### Advances in imaging

The rates of seizure freedom following resective surgery in sufferers of non-lesional extratemporal epilepsy remain poor.^2^ It is clear that better patient selection is required in these cases, with improved methods for imaging the EZ and guiding the implantation of intracranial EEG. For some time there has been interest in unmasking previously occult structural lesions, using non-routine magnetic resonance (MR) sequences and voxel-based morphometric analyses.^5^ There are also developmental techniques, such as EEG-fMRI, which require refinement and further evaluation in clinical practice.^6^

**Figure 1:**
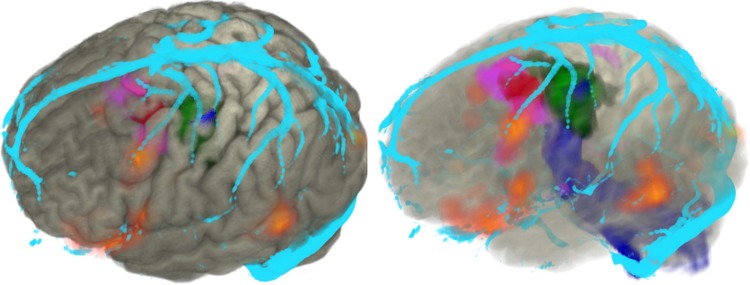
Volume rendering of cortex (grey) displayed in AMIRA with the following associated modalities: focal cortical dysplasia (red), fluorodeoxy glucose positron emission tomography (FDG-PET). hypometabolism (purple), hand motor fMRI (green), corticospinal tractography (blue), veins (cyan).

3D multimodality imaging is the simultaneous display of different structural and functional datasets, tailored to individual patients. The feasibility of this additional tool in a busy epilepsy surgery practice has been demonstrated,^7^ and a prospective study is currently underway to validate the usefulness of this in presurgical evaluation and surgical management. Ultimately, improvements in the surgical outcomes in this patient group will depend on better imaging, including visualisation of electrical abnormalities in 3D, reliable imaging integration and robust planning and implementation of intracranial EEG (figure 1).

## Neuroablation

There is an increasing trend in all forms of surgery towards minimally invasive techniques. This is most pertinent for neurosurgery, which often requires access to deep parts of the brain. Accurate navigation to these areas without the need for significant brain retraction has been solved by the application of stereotaxis. However, there remains the problem of how to ‘execute’ the surgery once instruments have been safely navigated to their targets. This problem is best framed in the context of epilepsy surgery with cortical resection following SEEG. However, it is also possible to consider patients with hippocampal sclerosis (HS), where even ‘selective’ amygdalohippocampectomies carry the risk of new cognitive deficit.

There are a number of interesting alternatives to ‘execute’ lesioning at the site of the EZ or to cause a disconnection in a minimally invasive way.

### Radiofrequency (RF) thermocoagulation

There is much interest in complementing the technique of SEEG with a therapeutic component to lesion cortex that is sampled by the electrodes. The most obvious solution is by thermocoagulation, using a RF generator connected to the electrode contacts. A feasibility study from Lyons described this technique in 2004, in 20 patients undergoing SEEG implantation.^8^

There are several benefits with this technique. It builds on the SEEG method that is well established, and proven to be safe and reliable. It is well tolerated by the patient and does not require general anaesthesia. Multiple sites can be lesioned, with real-time clinical and electrophysiological feedback. Finally, this method does not preclude the possibility of subsequent conventional open surgery.

One disadvantage with this technique is that there is no real-time feedback on the lesioning process with regards to local temperatures. The operator relies on an abrupt decrease in current to indicate coagulation of surrounding cortex. Also, RF thermocoagulation is known to be an inherently imprecise mode of thermal energy delivery, with theoretical risk to surrounding structures.

Overall, the results for SEEG and thermocoagulation are modest. A case series of 41 patients from the Lyons group report that 20 (48.7%) experienced a significant decrease in seizures of at least 50%, and 21 (51.3%) did not benefit from the procedure.^9^ Only one patient was seizure-free following the procedure. There were no reports of worsening seizures. These results suggest that RF thermocoagulation may be a low-risk, palliative procedure, which can be considered as first-line treatment in patients undergoing SEEG to improve seizure control. It seems that this technique is particularly suited to patients for whom conventional surgery is contra-indicated or considered too high risk, such as patients with deep epileptogenic heterotopic nodules. However, a randomised controlled trial is needed to determine which patient group exactly is most likely to benefit from RF thermocoagulation.

### MR-guided focused ultrasound

Magnetic resonance-guided focused ultrasound surgery (MRgFUS) is an accurate method of delivering high doses of transcranial ultrasound energy to a discrete intracranial focal point.^10^ The major historical barrier to this method was the need to create a craniectomy window prior to treatment, to prevent the ‘defocusing’ effect of the skull. However, recent advances in phased array transducer technology have overcome this defocusing effect, so that the treatment can be administered in a ‘closed’ method without the need for conventional surgery.

**Figure 2:**
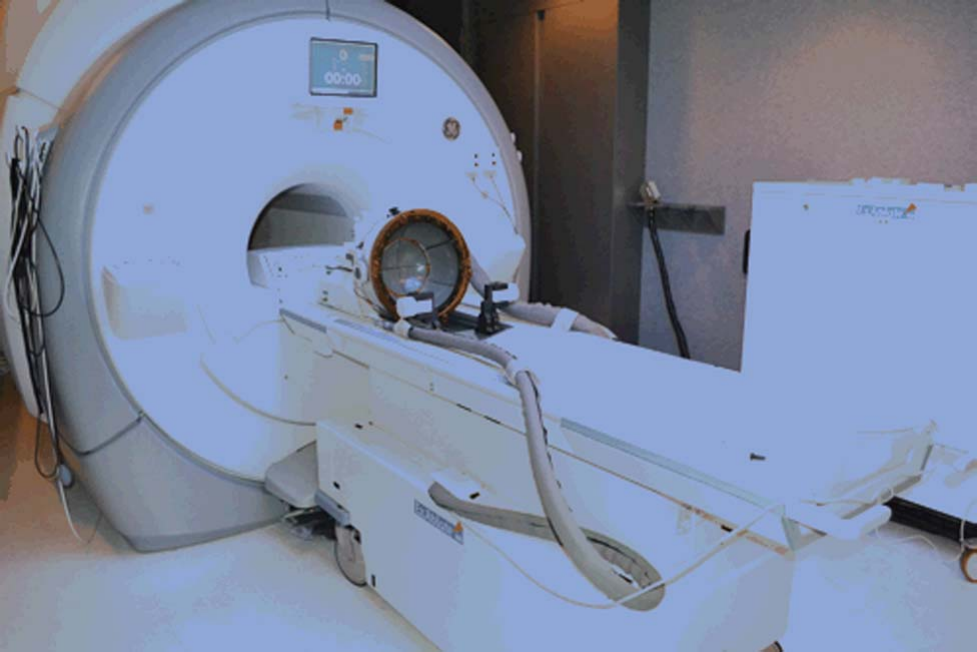
Commercially available magnetic resonance-guided (MR-guided) focused ultrasound.^10^

The MRgFUS consists of a clinical 3 T MRI, with a transcranial hemispheric array transducer that has 1024 ultrasound elements. The patient’s head is fixed to the system in a stereotactic frame and the transducer is filled with degassed water to allow ultrasound waves to propagate toward the patient's head. Treatment planning is based on MRI, and MR thermometry is used for target verification during the procedure. The treatment can be administered on an outpatient basis, and treatment effect can be monitored by postoperative MRI (figure 2).

MRgFUS has previously been used to execute a selective medial thalamotomy in the treatment of chronic neuropathic pain.^11^ A US food and drug administration (FDA)-approved phase I trial using MRgFUS thalamotomy in the treatment of essential tremor has just been completed, and showed clinical improvements in 15 patients.^12^ There are plans for further trials in the treatment of metastatic brain tumour and Parkinson's disease.

MRgFUS has obvious and compelling attractions in epilepsy surgery. There is the avoidance of any latency period or the risk of secondary tumours with ionising radiation, which comes with radiosurgery. There is the convenience of the treatment, which does not involve any skin incision and, therefore, avoids the surgical risks of infection, haemorrhage and wound dehiscence. There are no trajectory restrictions and, crucially, there is near-real-time feedback of the lesioning effect, with MRI thermometry.

The main concern with MRgFUS is the risk of inadvertent heating of the skull base and critical structures such as cranial nerves, which results from the ‘shadow’ effect of energy distal to the focal point of the target. Cadaveric studies have yielded techniques to minimise this collateral heating, by building into the system software certain ‘no pass’ areas at the base of the brain.^13^ However, this remains a significant barrier (at present) to the use of MRgFUS to lesion cortical and subcortical targets. In theory, MRgFUS should evolve to become an important treatment modality in epilepsy surgery, although it is important to note there are no current cadaveric or clinical trials underway to determine efficacy.

### Laser ablation

Ablation can also be achieved by MRI-guided laser interstitial thermal therapy (MRgLITT). The commercially available Visualase Thermal Therapy System combines a 15W 980 nm diode laser and cooled laser application system with an image-processing workstation. The applicator is inserted to reach the target by a stereotactic method, and laser treatment is applied in the MR scanner, with MR thermal imaging to visualise the thermal ablation.

MRgLITT avoids the complications associated with radiosurgery. The ablation is more precise than that achieved with RF thermocoagulation, and has reliable real-time feedback. Furthermore, it appears to avoid the heating of the skull base seen in ultrasound ablation. MRgLITT is a stereotactic surgical procedure, however, and therefore carries the surgical risks of haemorrhage and infection.

This technology has recently received FDA clearance for ablation in neurosurgery, and has previously been reported in the treatment of brain metastases.^14^ A recent study describes the initial use of this technique in the treatment of focal epilepsy in five children.^15^ Lesions included a cingulate tuber, HS, hypothalamic hamartoma (HH) and focal cortical dysplasia. There were no complications, and early experience indicates that this is a safe procedure. All patients were seizure-free at the time of going to print, but follow-up is short and no meaningful information can be drawn on long-term efficacy at present. A pilot study is currently underway, which will examine longer-term seizure outcomes in 20 patients, It is our view that MRgLITT is an exciting prospect, which is closer to clinical adoption in epilepsy surgery than is MRgUS.

### Stereotactic radiosurgery

Stereotactic radiosurgery (SRS) is a well-established technique that uses focused ionising radiation to target deep-seated lesions, sparing damage to surrounding tissue. The ionising radiation breaks chemical bonds and results in the production of free radicals. Ionising radiation can be generated by proton beam accelerators and photon accelerators. The most widely used sources of ionising radiation are photon accelerators, such as Cyberknife and Gamma Knife.

The main advantage of SRS is that deep-seated and multiple lesions can be treated without any surgical approach, avoiding the inherent brain retraction/injury. The main disadvantages include the latent period of efficacy, collateral tissue injury secondary to radiation, and late-onset secondary malignancies. Furthermore, the efficacy of SRS in the treatment of different conditions is not fully understood.

The anticonvulsant effects of SRS were first observed in the treatment of tumours and vascular lesions.^16^ SRS has also been used as a disconnection technique in the treatment of generalised epilepsy by corpus callosotomy.^17^ Current interest is centred mainly on the treatment of HHs and HS.

### Hypothalamic hamartoma

SRS is increasingly being considered for the treatment of HH. Treatment is best done in early childhood, before the development of secondary generalised seizures, behavioural problems and developmental delay. In selected cases, conventional open surgery offers higher rates of seizure freedom, with the pooled results from multiple studies showing seizure freedom rates from open surgery at 50% and SRS at 30–40%.^18^–^20^ However, depending on the characteristics and location of the HH, open surgery may be considered too hazardous, and SRS is the obvious alternative.^21^ SRS can also be used in conjunction with open surgery in patients with large HH, if surgical debulking leaves an unresectable residual epileptogenic intrahypothalamic component.^22^

### Hippocampal sclerosis

The use of SRS in the treatment of HS is controversial, since conventional anterior temporal lobe resections offer a proven and reliable treatment method. The theoretical advantage of SRS is that the EZ may be lesioned in a selective way, without injury to the lateral neocortex and corresponding neuropsychological complications.

The results of an early prospective multicentre trial on the efficacy of SRS in the treatment of MTLE were promising, with seizure outcome at 2 years comparable to that of standard surgery.^23^ No significant cognitive deficits were seen and, in fact, 20% experienced some degree of cognitive improvement. This compares favourably to standard surgery in which cognitive impairment, particularly memory and word-finding, is observed in 30%, and improvements are seen in 10–20%.^24^

A further multicentre prospective trial in the USA randomised patients to SRS with high (24 Gy) or low (20 Gy) dose delivered to the targets.^25^ At 3 years, seizure freedom was 77% in the high dose and 59% in the low dose group. Again, the neuropsychological profiles compared favourably with results from conventional surgery.

Despite these results, there remain questions on the use of SRS in MTLE. There are a number of published series that do not give the same efficacy rates in terms of seizure outcome.^26–28^ Interpretation of these is complicated by differences in protocol, including dose, isodose centres or ‘shots’, and volume, but the discordance in results should not be ignored.

The rate of optic radiation injury appears to be similar to that seen in conventional open surgery, with homonymous field defects seen in 43–50%..^23^ ^25^ This is a significant risk in those patients who become seizure-free and aspire to gain a driving license, and may compare poorly with the results of open surgery in which intraoperative visualisation of optic radiation tractography may be employed^29^ and reduce the risk of visual field defects.

Finally, there are unique risks with SRS that are not seen with conventional surgery, which may be related to the latent time course. Progressive radiological changes are observed, with the development of dose-dependent T2 hyperintensity, contrast enhancement and vasogenic oedema, with mass effect at 9 months postoperatively, and peaking at 12 months. These changes correspond to declines in complex partial seizures and transient increases in auras; 70% of patients in the Barbaro study also report new onset headaches postoperatively, although the timing of these is not predictable.^25^

A National Institutes of Health (HIF)-funded multicentre randomised controlled trial, the Radiosurgery or Open Surgery for Epilepsy (ROSE) trial, was designed to answer these outstanding questions. The trial randomised patients with MTLE to conventional surgery or SRS, and was to compare seizure outcome, cognitive outcome, quality of life and cost with an initial 3-year follow-up. Unfortunately, recruitment to this trial has currently stopped, with poor recruitment cited as the reason, and the continued funding of the work is in doubt. Without class 1 evidence, the relative merits of these two treatment paradigms will likely remain unclear.

### Extratemporal epilepsies

There are no reports on the use of SRS in non-lesional extratemporal epilepsy. Certainly, the prerequisite need for intracranial implantation to determine the EZ would negate the main benefit of SRS as a non-invasive procedure. However, since other ablative methods have their own disadvantages, and so far produce only modest results, SRS should not be completely dismissed as a possibility.

## Neuromodulation

Functional neurosurgery refers to the surgical manipulation of brain behaviour by the stimulation or removal of a population of neurones. The most successful application of functional neurosurgery is in the stimulation treatment of movement disorders such as idiopathic Parkinson's disease and essential tremor. However, there are also applications for chronic pain disorders, psychiatric disorders and epilepsy.

In this context, resective epilepsy surgery may be considered under the umbrella term, functional neurosurgery, since it involves the removal of a population of cells in the EZ. However, conventionally functional neurosurgery in epilepsy refers to the stimulation of cell populations, either through a cranial nerve or directly through an implanted electrode.

### Cranial nerve stimulation

#### Vagal nerve stimulation

Vagal nerve stimulation (VNS) is a well-established palliative treatment for epilepsy, in patients who are not candidates for resective surgery. Although VNS is unlikely to offer any advance in epilepsy surgery, the elucidation of the mechanism of action may have important consequences for other related treatments. Current evidence points towards a deactivation of the nucleus of the solitary tract, with widespread projections to the dorsal raphe nucleus, locus coeruleus, hypothalamus, thalamus, amygdala and hippocampus.^30^

#### Trigeminal nerve stimulation

Trigeminal nerve stimulation (TNS) is similar to VNS as it involves the widespread stimulation of brain nuclei from an afferent cranial nerve. Like VNS, the mechanism of action is not well understood, but is thought to involve deactivation of the nucleus of the solitary tract and the locus coeruleus.

TNS differs from VNS in several important ways. First, TNS does not require surgical implantation, but can be delivered in a flexible and disposable way by application of electrodes to the forehead to stimulate the supra-orbital nerves. This confers a significant advantage, offering patients a trial of treatment to assess whether they are likely to be responders, without exposure to the surgical risks of implantation. This also opens up the opportunity for structural and functional MR studies in patients undergoing TNS.

Second, the type of stimulation offered by TNS differs from VNS, as it is high frequency and bilateral. In animal models, this type of stimulation has been shown to be superior, with increased magnitude of the seizure-reduction effect.^31^

A randomised controlled trial of TNS in drug-resistant epilepsy has provided preliminary evidence that this is a safe and effective treatment, with a significant within-group improvement in responder rate over time, defined as a 50% reduction in seizure frequency seen at 18 weeks.^32^ However, no significant differences were seen between groups, due to the high responder rate in the control group. No patients were rendered seizure-free with this treatment. Large-scale phase III multicentre trials are required to better understand the efficacy of TNS, and also to optimise treatment parameters. There are a number of variables that can be manipulated, including frequency, amplitude and duration of stimulation. Despite this, the future role for TNS is likely to be as a more tailored, safer alternative to VNS; it may offer the twin benefits of improved seizure control and improvements in mood, but is unlikely to be a curative treatment.

### Deep brain stimulation

There is a long history of interest in the use of deep brain stimulation (DBS) for epilepsy control. The postulated mechanism of action is by interrupting the propagation of seizure activity, or by increasing the overall seizure threshold. Multiple targets have been put forward, centred in and around the circuit of Papez. Wu *et al* have provided an excellent summary of DBS targets for the treatment of epilepsy^33^ (table 1)

**Table 1:** Table to show the summary of targets for DBS for the treatment of epilepsy

	Controlled trial		
Target	Primary author	Year	Outcome
Anterior nucleus of the thalamus	Fisher	2010	SANTE trial: 40.4% median seizure reduction
Cerebellum	Van Buren	1978	69% seizure reduction in 80% patients
	Wright	1984	Ineffective
	Velasco	2005	>50% seizure reduction in 80% cases
Centromedian nucleus of the thalamus	Fisher	1992	>50% seizure reduction in 80% cases
	Velasco	2000	No significant difference
Hippocampus	Tellez-Zenteno	2006	15% seizure reduction
	Velasco	2007	>50% seizure reduction in all 9 patients
	McLachlan	2010	33% seizure reduction
Caudate nucleus	None (case reports only)		
Subthalamic nucleus	None (case reports only)		
Corpus callosum/fornix	None (animal models only)		
Posterior hypothalamic mammillary nuclei	None (case reports only)		
Locus coeruleus	None (case reports only)		

Amended from Wu and Sharan.^33^

DBS, deep brain stimulation.

.

The current results with DBS for the treatment of epilepsy remain modest, even accounting for the difficult patient group with highly refractory epilepsy. Stimulation-related side effects have been reported, most commonly with psychiatric disturbances and depression. There is also the possibility of habituation to long-term stimulation. Taken together, these considerations have stimulated interest in a closed-loop, responsive form of neurostimulation, which only administers stimulation if triggered by seizure activity.

### Closed-loop stimulation

The mechanism of action for closed-loop stimulation (CLS) is similar to DBS, with a rising of seizure threshold or inhibition of propagation. Targets for stimulation depend on the accurate localisation of the EZ, which is a prerequisite to implantation. This technique is most appropriate for patients with bitemporal or multifocal epilepsy, with no more than two distinct EZ. It also complements the technique of SEEG in cases where the EZ is localised to eloquent cortex with considerable risks with resection.

CLS relies on a robust form of automated seizure detection. The most popular methods are based on analysis of EEG waveforms across the time domain, and include the following:

- line length tool, to detect changes in frequency
- area tool, to detect changes in signal amplitude
- half-wave tool, to detect the number of waves that exceed a predetermined amplitude and duration.

There does not appear to be any single superior method for seizure detection. Instead, a tailored approach seems most promising, using combinations of these tools modified and optimised for individual patients.^34^

The RNS System Pivotal Clinical Investigation was a multicentre, randomised, double-blind sham stimulation controlled trial in 191 patients with refractory epilepsy^35^ During the initial 12-week blinded evaluation period, an initial implantation effect was noted, with a reduction in seizure frequency in the stimulation and sham groups of 34.2% and 25.2%, respectively. This implantation effect was thought to be a placebo effect, or an effect of the anaesthesia. However, as the blinded period progressed, the improvement in seizure frequency persisted and improved in the stimulation group to 41.5%, while improvement in the sham group fell by 9.4%. At 2 years median, seizure frequency was reduced by more than 50% with a responder rate of greater than 45% with stimulation. Additionally, there were significant improvements in quality of life reported by patients receiving stimulation, via the Quality of life in Epilepsy Inventory (QOLIE-89). Of particular interest, there was no reported worsening of neuropsychological conditions or mood disorders that were commonly seen in conventional DBS.

**Figure 3:**
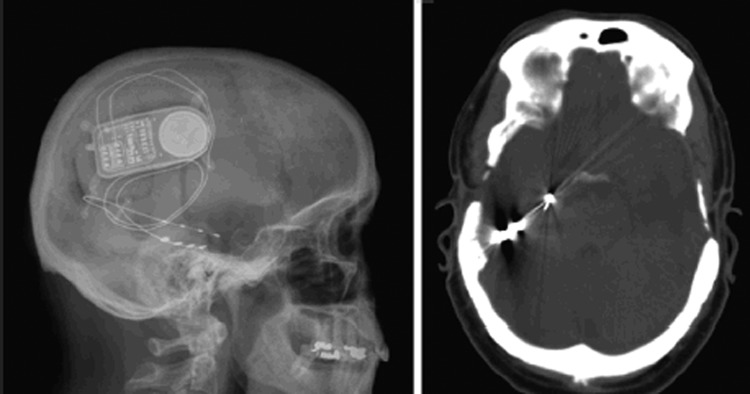
Closed-loop stimulation, with a depth electrode in the right hippocampus and a subdural grid over the inferior surface of the temporal lobe.^35^

Clearly, CLS requires more study, with outstanding questions on seizure detection algorithms, targets and stimulation parameters, and long-term follow-up. However, this remains an exciting prospect as a further treatment modality in refractory epilepsy (figure 3).

### Closed-loop local drug delivery

It is an attractive possibility that localised intracerebral delivery of antiepileptic drugs (AED) can improve the efficacy of pharmacological treatment of epilepsy, without systemic side effects^36^ Several groups are, therefore, engaged in research developing automated local drug delivery systems, comprising of seizure detection technology coupled with intracranial delivery of AED^37^ The most promising of these is the subdural hybrid neuroprosthesis (HNP), described by Ludvig. Safety and efficacy is currently being investigated in rat and monkey models, but there remains some distance to go before human clinical trials. Conceptually, this is closely related to the closed-loop systems of neurostimulation. However, this is arguably a more challenging path to take, requiring close collaboration between the disciplines of microengineering, neuropharmacology, neurophysiology and neurosurgery.

## Summary

There are several exciting avenues for further technological advances in the surgical treatment of epilepsy surgery.

For patients undergoing open surgery, improving their outcomes will depend on the stepwise refinement of current methodology, with advances in imaging epilepsy paramount. For patients who are currently not candidates for open surgery, increased delivery of treatment options by minimally invasive techniques, either neuromodulatory or ablative in nature, is likely to occur in the future.

Advances in neuromodulation will coincide with the elucidation of the mechanism of action, and much work needs to be done to refine individual stimulation targets and stimulation parameters. New neuromodulation techniques will also require a robust evidence base for clinical indications, long-term efficacy and safety. As described, there are real difficulties in designing trials for new epilepsy treatments where the findings of high responder rates in control groups are a common feature and compromise the interpretation of studies. One reason for control responses is the well-documented placebo effect, which is often present following functional sham surgery. Other possible causes, seen in drug trials and trials of new surgical treatments, are related to the selection of the control group. The group can be compromised by the phenomenon of ‘regression towards the mean’, where inconsistency in seizure frequency, and high initial seizure frequency, can later give the false impression of response rates in the control group as the study progresses. Additionally, it is not uncommon for patients in control groups to have their medical treatment optimised, with a consequent improvement in seizure control. Finally, outcome is a complex term in epilepsy surgery, encompassing the interplay of seizure freedom, seizure control, cognitive and surgical morbidity and quality of life over a prolonged period of time. In the future, large-scale and well-designed randomised controlled controls will have to take these factors into account to provide the evidence base for clinical implementation.

There is no consensus on the most promising technique for neuroablation, and competition between different methods will continue. In terms of clinical implementation, there are already centres that use SRS and SEEG-guided RF routinely, while MRgLITT is undergoing early clinical trials and MRgFUS remains very much a research tool in epilepsy. One major disadvantage of neuroablation in general is that the size of the lesioning is limited, and large, effective ‘resections’ can only be achieved by the repeated lesioning of different contiguous targets. For SEEG-guided RF and MRgLITT, this entails repeated passage of hardware through the brain, with associated risk of vascular injury. The precise targeting of individual propagation pathways and epileptogenic foci is, therefore, likely to be a largely palliative measure, with the eventual emergence of previously masked pathways and foci to continue seizure propagation following surgery. Unfortunately, this may be an insurmountable limitation with neuroablation when compared with conventional open surgery and cortical resection.

Finally, epilepsy surgery remains a significantly underused resource. It is often perceived as a treatment of last resort, with patients typically referred after 20 years of seizures.^38^ This contrasts with the NICE (National Institute of Health and Care Excellence) guidelines, which recommend referral to a tertiary service if epilepsy is not controlled within 2 years (NICE clinical guideline 137, 2012).^39^ This also runs counter to the evidence that epilepsy surgery is a cost-effective treatment, with large savings in seizure-free patients, as anticonvulsants and hospital admissions are successfully eliminated.^40^ Perhaps the most important advance for the future would be to increase awareness in the general population, and education among health professionals, on the safety and efficacy of epilepsy surgery as an early intervention in medically refractory focal epilepsy.

Early referrals to tertiary centres, coupled with the rigorous application of systematic presurgical evaluation pathways in a multidisciplinary environment, and with 3D multimodality imaging, may be the simplest and surest way to advance epilepsy surgery in the near future.
